# Landscape of Alternative Splicing Events Related to Prognosis and Immune Infiltration in Glioma: A Data Analysis and Basic Verification

**DOI:** 10.1155/2022/2671891

**Published:** 2022-07-04

**Authors:** Hong-xin Su, Gang Yang, Fei Su, Chen-xiao Hu, Tao Zhang, Jun-tao Ran, Quan-lin Guan

**Affiliations:** ^1^Department of Radiation Oncology, The First Hospital of Lanzhou University, Lanzhou City, Gansu 730000, China; ^2^Department of Neurosurgery, The First Hospital of Lanzhou University, Lanzhou City, Gansu 730000, China; ^3^Department of Oncology, The First Hospital of Lanzhou University, Lanzhou City, Gansu 730000, China; ^4^Information Centre, The First Hospital of Lanzhou University, Lanzhou City, Gansu 730000, China; ^5^Department of Oncology Surgery, The First Hospital of Lanzhou University, Lanzhou City, Gansu 730000, China

## Abstract

**Background:**

Glioma is a prevalent primary brain cancer with high invasiveness and typical local diffuse infiltration. Alternative splicing (AS), as a pervasive transcriptional regulatory mechanism, amplifies the coding capacity of the genome and promotes the progression of malignancies. This study was aimed at identifying AS events and novel biomarkers associated with survival for glioma.

**Methods:**

RNA splicing patterns were collected from The Cancer Genome Atlas SpliceSeq database, followed by calculating the percentage of splicing index. Expression profiles and related clinical information of glioma were integrated based on the UCSC Xena database. The AS events in glioma were further analyzed, and glioma prognosis-related splicing factors were identified with the use of bioinformatics analysis and laboratory techniques. Further immune infiltration analysis was performed.

**Results:**

Altogether, 9028 AS events were discovered. Upon univariate Cox analysis, 425 AS events were found to be related to the survival of patients with glioma, and 42 AS events were further screened to construct the final prognostic model (area under the curve = 0.974). Additionally, decreased expression of the splicing factors including Neuro-Oncological Ventral Antigen 1 (NOVA1), heterogeneous nuclear ribonucleoprotein C (HNRNPC), heterogeneous nuclear ribonucleoprotein L-like protein (HNRNPLL), and RNA-Binding Motif Protein 4 (RBM4) contributed to the poor survival in glioma. The immune infiltration analysis demonstrated that AS events were related to the proportion of immune cells infiltrating in glioma.

**Conclusions:**

It is of great value for comprehensive consideration of AS events, splicing networks, and related molecular subtype clusters in revealing the underlying mechanism and immune microenvironment remodeling for glioma, which provides clues for the further verification of related therapeutic targets.

## 1. Introduction

Genomic analysis has become an important tool for developing new therapeutic options, which has facilitated the opening of a new era of tumor genomics research. The mRNA precursors are spliced, and the remaining exons can be reconnected in different ways at the posttranslational level. The resultant diverse mature mRNAs are then translated into different protein variants. The process is defined as alternative splicing (AS), and over 90% of human multiexon genes undergo AS [1]. Protein factors involved in the splicing process of RNA precursors are considered splicing factors (SFs). Consequently, a gene can encode multiple proteins, which contributes to proteome diversity and cell complexity [2].

AS plays a critical role in numerous vital biological processes, including tissue differentiation; its dysfunction may induce multiple diseases, such as neurodegenerative diseases [3], malignant tumors [4], bladder cancer [5], lung cancer [6], ovarian cancer [7], gastric cancer [8], colorectal carcinoma [9], breast cancer [10], oral squamous cell carcinoma [11], and lymphoma [12]. Previous studies [2] have confirmed that a number of genes have tumor-specific splice variants, which share different functions from the typical variants expressed in normal tissues. These gene variants participate in multiple key pathways related to tumorigenesis, such as apoptosis regulation, DNA repair, proliferation, and migration. Splicing defects can also lead to genomic instability, which is a common feature of tumors, as well as being the driver of tumor progression. Recently, accumulating evidence demonstrated that AS contributed to the tumor microenvironment remodeling by affecting the proportion of immune infiltrating cells and regulating the activity of immune cells [13, 14]. Thus, cancer-specific AS might serve as a potential biomarker.

Glioma is a tumor that originates from neuroglial cells in the brain. According to the standard formulated by the World Health Organization (WHO), glioma is classified as grade I, II, III, or IV [15]. Typically, low-grade gliomas (grades I and II) have low invasiveness and relatively favorable prognosis, but they usually cause damage to the functional areas of the brain, whereas high-grade gliomas (grades III and IV) are known to be aggressive, with poor survival. The average survival of patients with glioma is about 15 months, and the 5-year overall survival (OS) rate is <5% (WHO 2016). Currently, there are only a few reliable biomarkers for predicting the course or prognosis of patients with glioma [16]. Due to the limited treatment methods and poor curative effect of glioma, especially high-grade glioma, there is an increased need to find more efficient targets.

Glioma exhibits significant heterogeneity at microscopic and molecular levels. The Cancer Genome Atlas (TCGA) can provide an integrated somatic cell atlas for glioma based on molecular and clinical data, which is a valuable resource for investigating genomic disorder in glioma. RNA processing is a major contributor to transcript variation and gene expression regulation and plays a pivotal role in many diseases and cancers. Therefore, it is extremely necessary to systemically analyze AS and its regulations for tumor immune microenvironment in glioma.

## 2. Methods

### 2.1. AS Events in TCGA RNA-seq Database

The mRNA splicing profiles were extracted from TCGA SpliceSeq (https://bioinformatics.mdanderson.org/TCGASpliceSeq/) database. The percentage splicing index (PSI) (range, 0–100%) is used to quantify AS events and calculate seven AS types: Mutually Exclusive Exons (ME), Exon Skip (ES), Retained Intron (RI), Alternate Terminator (AT), Alternate Promoter (AP), Alternate Acceptor site (AA), and Alternate Donor site (AD). In this study, PSI > 80% and minimum PSI standard deviation > 0.1 were established as the thresholds to screen the splicing patterns of related protein-coding genes in patients with glioma. In addition, UCSC Xena (http://xena.ucsc.edu/) was utilized to obtain the glioma expression profile data and related clinical information, which were later integrated with AS data for further investigation.

### 2.2. Survival Analysis

Patients were classified into two groups based on the median threshold of every parameter. AS events were subjected to univariate and multivariate Cox regression analyses to identify their association with OS, and *p* < 0.05 was used to indicate significant associations. A prognostic risk score was calculated by multiplying the AS PSI linear combination by the related regression coefficient (*b*) that indicated related weight. In this study, the regression coefficient was determined using the multivariate Cox proportional hazard regression model. The risk score formula is shown below:

(1) $$\begin{array}{c} \text{Risk Score}=\text{PSI of }{\text{AS}}_{1}\times b_{{\text{AS}}_{1}}+\text{PSI of }{\text{AS}}_{2}\times b_{{\text{AS}}_{2}}+\cdots +\text{PSI of }{\text{AS}}_{n}\times b_{{\text{AS}}_{n}}. \end{array}$$

Finally, the AS events that were shown to independently predict prognosis were integrated to construct a prognostic prediction model. We also plotted the survival curve and receiver operating characteristic (ROC) curve for the establishment of prognostic models. Kaplan-Meier analysis was performed for survival analysis, which was compared using the log-rank test.

### 2.3. Construction of a Splicing-Related Network

Altogether, 67 human SF lists were generated from manual literature and database screening. The gene expression profile data of SFs were extracted from UCSC Xena for subsequent analysis. Survival analysis (*p* < 0.05) was applied for the identification of SFs related to prognosis. Spearman correlation analysis was performed to analyze the correlation between survival-associated SF gene expression and survival-associated AS PSI value, and *p* < 0.05 and ∣cor | >0.3 were set to indicate the significant difference. Moreover, Cytoscape (3.6.0 National Resource for Network Biology) was used to construct an AS event-SF interaction network.

### 2.4. Prognostic- and Subtype Analysis-Related Clusters

AS events varied considerably among individual samples. Thus, to obtain a reliable classification, this study utilized the unsupervised consensus method with R package Consensus Cluster Plus to identify the glioma molecular subtypes. We assessed the prognostic value of different clusters to determine relationships of subtypes with survival. To investigate the potential regulation in biological processes, cellular components, and molecular functions, the ClusterProfiler package was employed for Gene Ontology (GO) functional enrichment analysis with genes involved in survival-associated AS events. Additionally, the ReactomePA package was utilized for Reactome pathway enrichment analysis to identify the significant pathways.

### 2.5. Tissue Samples

To validate the expression of key SFs in glioma, 10 tumors were collected from patients diagnosed with glioma who underwent surgery at the First Hospital of Lanzhou University. Of the 10 patients with glioma, there were 6 men and 4 women. They ranged in age from 35 to 74 years old, with an average age of 55.3 ± 7.8 years. Normal brain tissues were collected from 10 patients whose brain tissues were partially removed due to head injury. The samples were obtained with informed consent from all patients. This study was approved by the Ethics Committee of the First Hospital of Lanzhou University.

### 2.6. Western Blotting

Total protein was extracted from frozen tissues, and protein concentration was measured. The protein sample was isolated by sodium dodecyl sulfate polyacrylamide gel electrophoresis and then transferred onto a polyvinylidene fluoride membrane. After the membranes were blocked in BSA (10%) for 2 h, the target antibody was applied for the incubation with membranes overnight at 4°C. Subsequently, TBST was used to wash the membrane three times for 10 min, followed by incubation with a goat anti-IgG secondary antibody at ambient temperature. Thereafter, the membranes treated with ECL photoluminescence solution were analyzed using ImageJ software. More details about the antibodies were shown below: the anti-Nova1 antibody (1 : 1000), anti-RBM4 antibody (1 : 800), and anti-HNRNPLL antibody (1 : 1200) were purchased from Abcam (Cambridge, MA, USA).

### 2.7. Immune Cell Infiltration Analysis

The TIMER database, as a platform, could provide in-depth analysis and visualization of tumor 5infiltrating immune cells for various cancers [17]. The correlation between key SF gene expression and infiltration levels of B cells, CD4+ T cells, CD8+ T cells, macrophages, neutrophils, and dendritic cells in glioma was calculated with the TIMER database. To further assess the relationship between the key SF genes and immune cell infiltration, the CBERSORT algorithm was applied, which could calculate the proportion of 22 immune cells.

## 3. Results

### 3.1. Overview of AS Events

**Figure 1:**
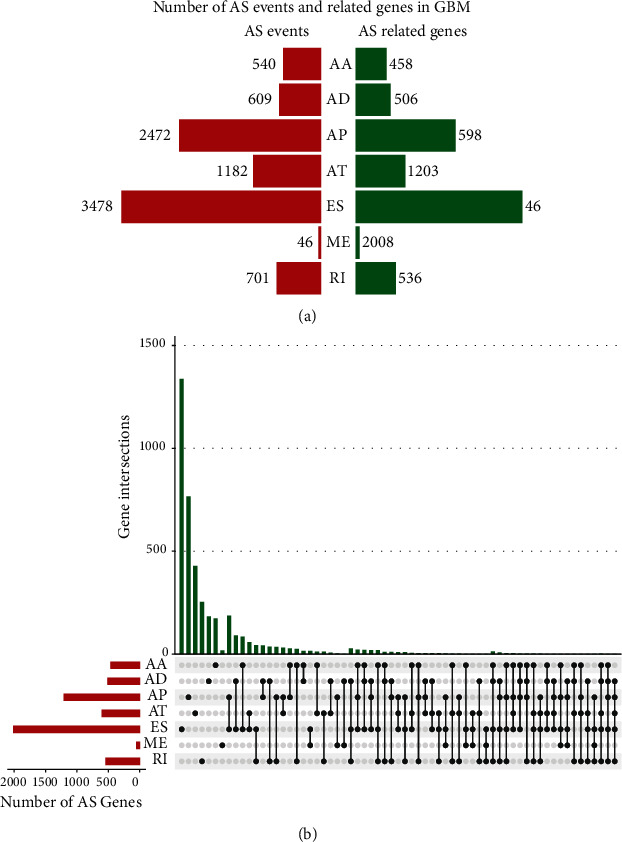
AS events in glioma: (a) various types of AS events and the number of genes involved; (b) UpSet diagram of the overall AS events involving interactions between genes.

In total, 9028 AS events were observed among 4117 genes in the 146 glioma samples based on TCGA database (Figure 1(a)). The findings indicated that a single gene was associated with multiple mRNA splicing event types, which underwent as many as five types of AS events. ES was the predominant AS event: over 1/3 AS events were ESs (Figure 1(b)).

### 3.2. Survival-Related AS Events in Glioma

**Figure 2:**
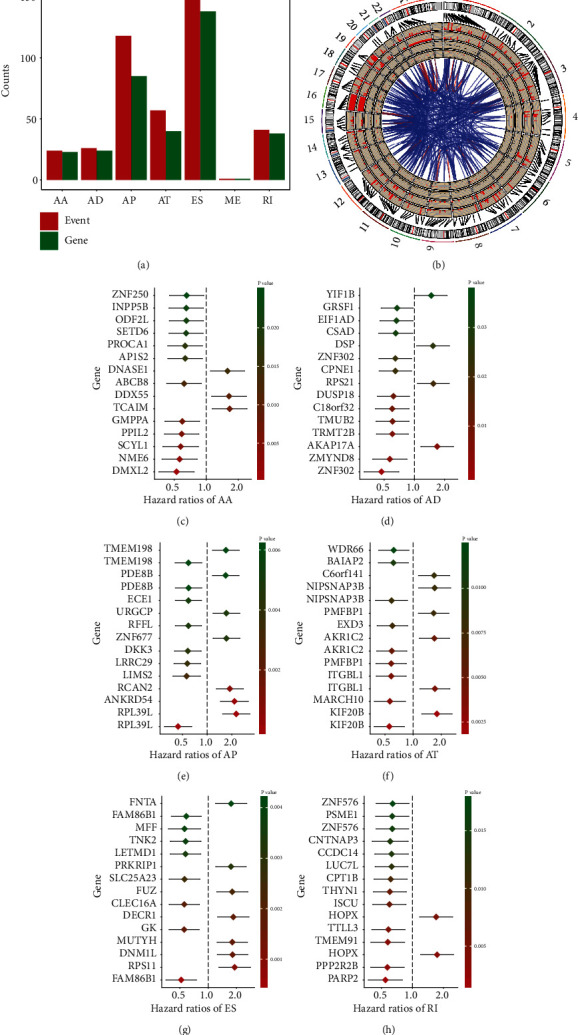
AS events and prognosis in glioma. (a) The number of survival-related AS events and related genes in each type of differential AS. (b) Circos diagram of survival-related AS events and related genes: the Circos panel from outside to inside is expressed as follows: chromosome number, genome axis, and survival-related AS event-related genes; the number of related genes in the overall AS event (showing 1-10 different heights, more than 10 calculated as 10); correlation; the number of AS types of genes in the overall event; the *p* values of related genes in the single-factor COX regression analysis (expressed by the conversion value of −log10 (*p* values); the higher the height, the more significant the *p* value); the HR value of correlation genes in the univariate COX regression analysis (in which red represents HR > 1 and black represents HR < 1); and the correlation between genes. (c–h) Forest diagram of top 15 AS events related to survival in each AS type.

To explore the significance of AS events in predicting the prognosis of glioma, univariate Cox regression analysis was performed. A total of 425 AS events were identified as significantly associated with survival (*p* < 0.05), including 24 AAs, 26 ADs, 188 APs, 57 ATs, 158 ESs, 1 ME, and 41 RIs (Figure 2(a)). As shown in Figure 2(b), the Circos graph displays the survival-associated AS events and the involved genes.

### 3.3. Construction of the Glioma Prognostic Models

**Figure 3:**
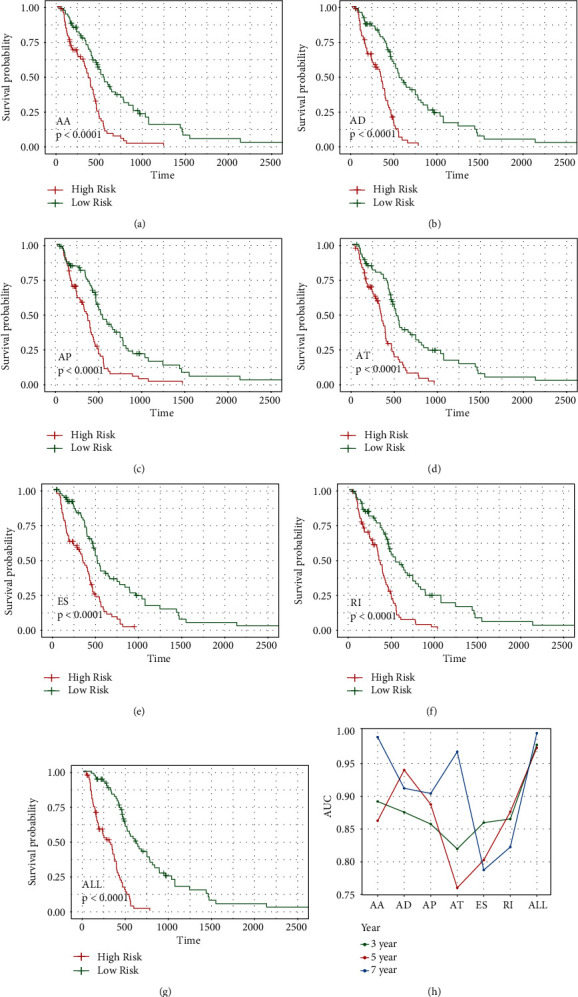
AS events and survival curve in glioma: (a–f) survival curve of prognostic factors related to each type of AS; (g) survival curve of the final AS prognostic model; (h) various AS types and the AUC value of the ROC curve of the final AS model in 3, 5, and 7 years.

The forest plot showed the top 15 prognostic AS events of the univariate analysis in each AS type (Figures 2(c)–2(h)) The top 15 AS events in each AS type were selected for multivariate Cox regression analysis and obtained 42 independent prognostic AS events, including AA events (*n* = 7), AD events (*n* = 8), AP events (*n* = 6), AT events (*n* = 5), ES (*n* = 10) events, and RI (*n* = 6) events. In total, the independent variables included 7 AA events, 8 AD events, 6 AP events, 5 AT events, 10 ES events, and 6 RI events. The prognostic AS events among the different AS types were incorporated to construct a prognosis model. Based on the analysis for every AS pattern, patients with a high risk score had lower survival rate compared to patients with a low risk score, as presented in Figures 3(a)–3(f), of which the model with AD events had the best accuracy in predicting 5-year survival (area under the curve (AUC) = 0.940) (Figure 3(h)). Moreover, when all possible AS events were included for independent prognosis prediction from the different types (total 42 AS events), the model displayed favorable ability in predicting the 5-year survival (AUC = 0.974), as presented in Figures 3(g) and 3(h).

### 3.4. Survival-Related AS-SF Interaction Network

**Figure 4:**
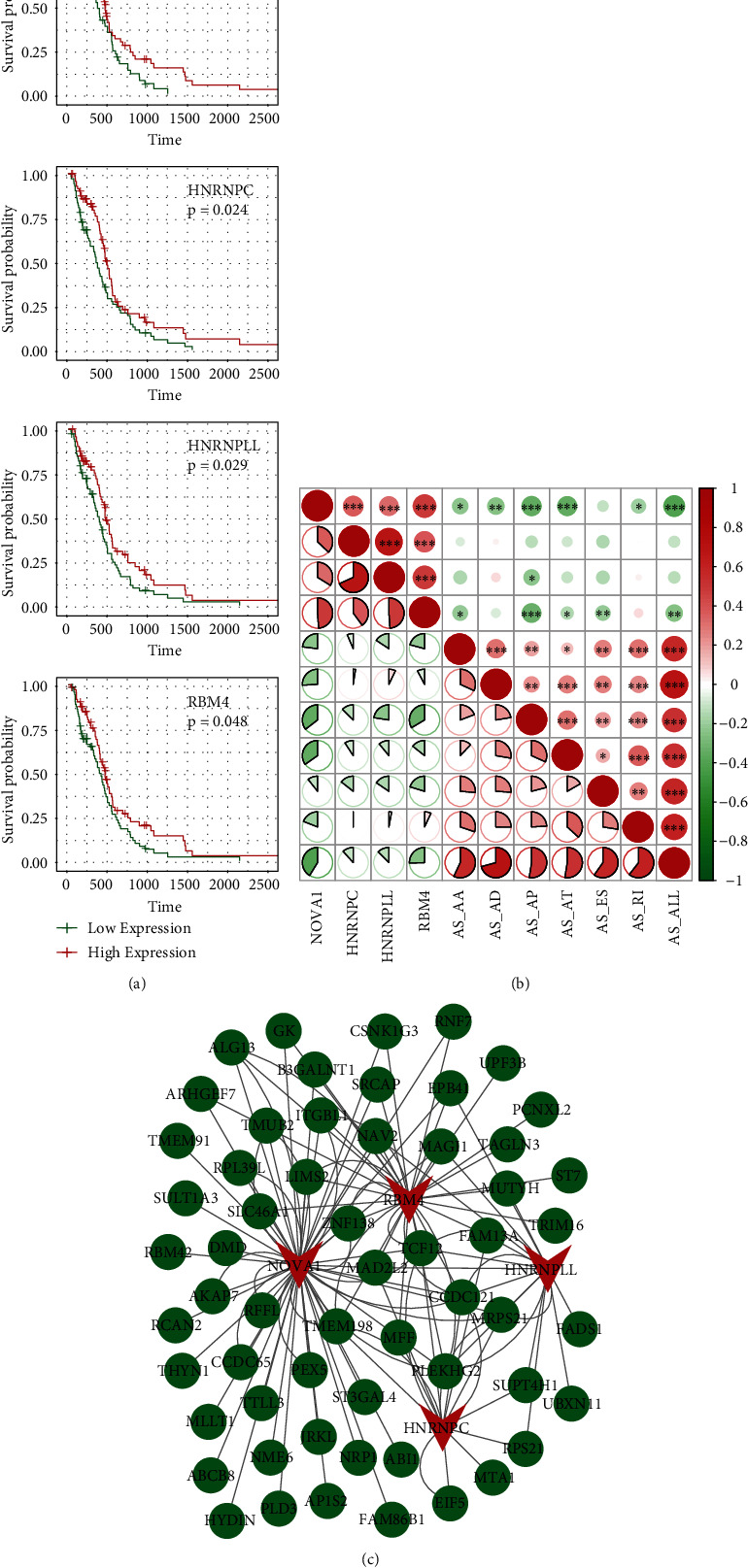
SFs in glioma. (a) Survival curve of SFs. (b) Correlation analysis of the splicing factor and AS prognostic predictor. The upper right figure shows the significance of the correlation between the correlation coefficient and SF expression and the PSI value of prognostic-related AS events, where ^∗^*p* < 0.05, ^∗∗^*p* < 0.01, and ^∗∗∗^*p* < 0.001; the lower left figure shows a pie chart of the correlation between SF expression and the PSI value of survival-related AS events, where the size of the pie chart represents the correlation, the green line represents the negative correlation, and the red line represents the positive correlation. (c) The AS network, where the green node represents the gene involved in the differential AS event and the red node represents the AS factor.

To determine the prognostic value of SFs, survival analysis was applied for SFs according to gene expression. As shown in Figure 4(a), four SFs exhibited a significant correlation with OS, and decreased levels led to poor prognosis. We also clarified the relationships between SFs and survival-associated AS events and established a prognostic model. Most SF gene levels were negatively correlated with the PSI (Figure 4(b)).

Additionally, the correlations between PSI values of survival-associated AS event and survival-related SF expression were investigated by Spearman correlation analysis (Figure 4(a)). Four survival-related SFs (red dots) showed a significant correlation between 58 genes (green dots) and survival-associated AS events. There were 59 associations of the SF NOVA1 and 35 associations of the SF RBM4 with AS events. In addition, as shown in Figure 4(c), *CCDC121*, *MAD2L2*, *TCF12*, and *ZNF138* interacted with all four SFs.

### 3.5. Prognosis-Related Molecular Subtype Clusters

**Figure 5:**
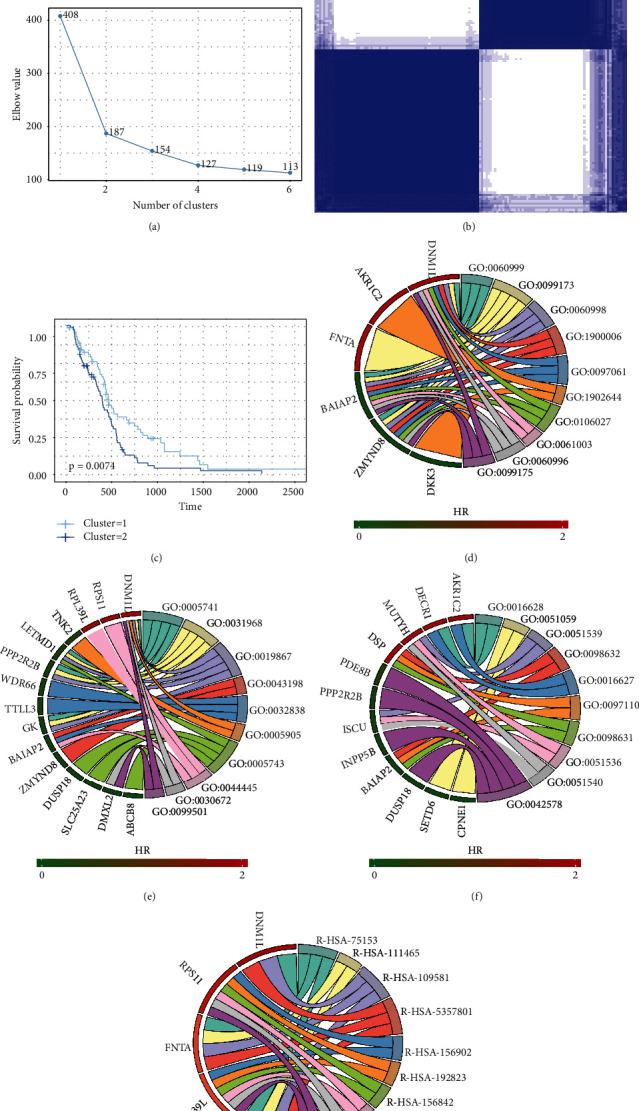
AS clusters associated with prognosis and molecular subtypes: (a) the elbow method identifies the optimal number of clusters; (b) the consensus matrix heat map defines two sample clusters, the consensus value of which ranges from 0 (white, samples never clustered together) to 1 (dark blue, samples always cluster together); (c) survival analysis in the two identified sample clusters; (d–f) top 10 significantly enriched biological processes, cell components, and molecular functions in GO analysis; (g) top 10 significantly enriched Reactome pathways.

To further identify different AS patterns, unsupervised analysis was conducted for all samples according to prognosis-related AS events. According to the results of the Consensus Cluster Plus analysis and the distribution of consensus values from 0 to 1, cluster 1 (*n* = 69, 47.3%) and cluster 2 (*n* = 77, 52.7%) were finally identified by the elbow method, as displayed in Figures 5(a) and 5(b). Subsequently, survival analysis was performed to evaluate the effect of these clusters on prognosis. The results showed that cluster 2 was related to poor survival, whereas cluster 1 was associated with favorable survival (Figure 5(c)). The related genes were significantly enriched in multiple GO terms and Reactome pathways, as presented in Figures 5(c)–5(g).

### 3.6. The Expression of Key SFs in Glioma

**Figure 6:**
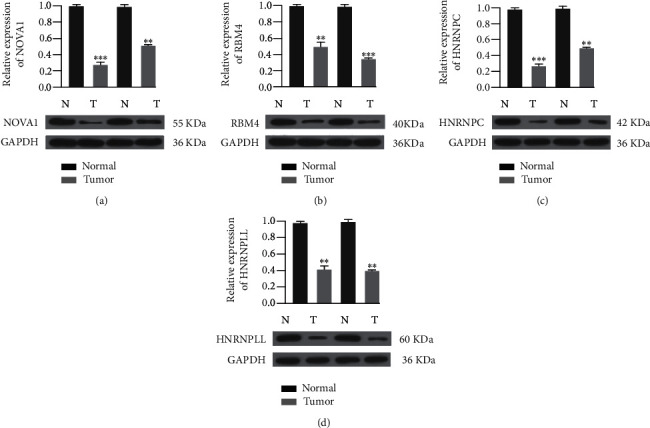
Western blotting detected the expression levels of four SFs in glioma and nontumor tissues. ^∗∗^*p* < 0.01, ^∗∗∗^*p* < 0.001.

To confirm the expression of key SFs in glioma, we conducted western blotting based on glioma and nontumor tissues. The results indicated that the expression levels of these four SFs, including NOVA1, RBM4, HNRNPC, and HNRPLL, in tumor tissues were significantly decreased, compared to those in normal tissues (Figure 6).

### 3.7. The Correlation between Key SFs and Immune Cell Infiltration

**Figure 7:**
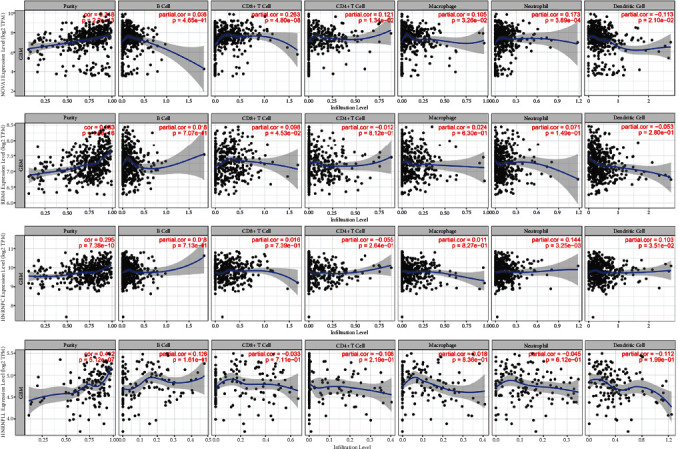
The correlation analysis between the expression of four SFs and immune cell infiltration in glioma based on the TIMER database.

**Figure 8:**
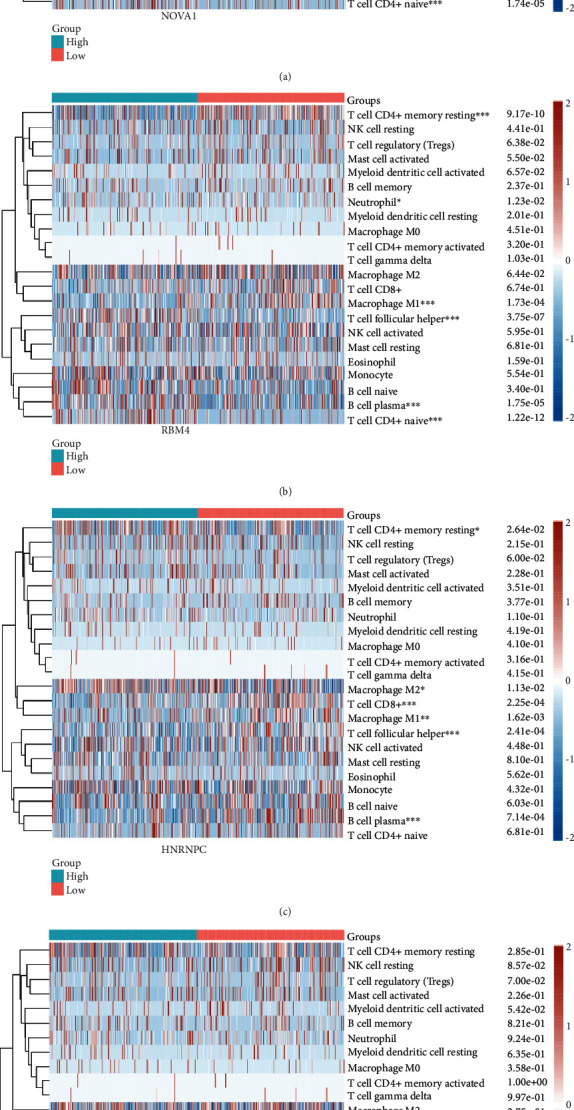
The difference of infiltrating immune cells between high and low gene expression with the CIBERSORT algorithm.

To calculate the correlation between the SF expression and immune infiltrates, the TIMER database was applied with the key SF expression in TCGA dataset. As presented in Figure 7, the expressions of NOVA1 and RBM4 were positively correlated with the infiltration of CD8+ T cells. Besides, NOVA1 expression was significantly related to the infiltration level of CD4+ T cells, macrophages, neutrophils, and dendritic cells. High expression levels of HNRNPC contributed to the infiltration of neutrophils and dendritic cells. Although there was no significant difference between HNRPLL expression and the infiltration of immune cells, HNRPLL was closely related to immune purity. Besides, the results of CBERSORT analysis were consistent with the results of the TIMER database (Figure 8). The above findings suggested that there might be an underlying correlation between the AS events and immune infiltration.

## 4. Discussion

In recent years, accumulated evidence suggested that AS occurred in multiple tumors and played a critical role in the progression of malignancies, including glioma. Our study found that genes involved in AS events affected glioma survival including NOVA1, HNRNPC, HNRNPLL, RBM4. Other scholars have discovered more influencing factors. Li et al. [18] verified that CX-5461 suppressed telomerase activity and induces cell apoptosis via the regulation of hTERT in glioma cells. In addition, it has been reported that the expression level of ELK1 exhibited the positive correlation with tumor malignant progression, which was regulated by AS [19]. Shao et al. [20] found that two variants of *ITSN1* via AS exercised distinctly different functions in glioma. Specifically, the short variant of ITSN1 promoted the development of glioma, whereas the long variant exerted a tumor-suppressive effect.

Previous studies examining AS have usually focused on a single gene or SF. Additionally, the prognostic value and immune infiltration analysis of AS in glioma have not yet been studied. To our knowledge, this study is the first to systematically identify and analyze AS events related to survival and immune cell infiltration in glioma. Based on our findings, 425 survival-related AS events were revealed, and SF genes related to survival-associated AS events were identified, including NOVA1, HNRNPC, HNRNPLL, and RBM4. These key SF genes were significantly related to the infiltration of immune cells in the tumor microenvironment. These factors exert vital roles in the genesis and development of multiple tumors [11, 21–32].

Among these four SFs, HNRNPC, a small nuclear ribonucleoprotein particle protein factor, is the most extensively investigated. Fischl et al. [23] reported that the HNRNPC-dependent alternative cleavage and polyadenylation (APA) profile changes in colonic tumors compared to noncarcinoma colonic epithelial cells. They also verified that HNRNPC is a key regulator of physiology-related APA events and postulated that HNRNPC might have facilitated gene expression related to proliferation and metastasis and promote carcinogenesis. The findings of Zhang et al. [24] demonstrated that HNRNPC interacted with the lncRNA *LBX2-AS1* and transcription factors ZEB1 and ZEB2 related to the epithelial-mesenchymal transition (EMT), which further facilitated the progression of EMT. Wen et al. [10] discovered that HNRNPC could serve as a predictor for tumor burden and prognosis. Additionally, high expression level of HNRNPC contributed to poor survival of patients with gastric cancer receiving chemotherapy and resulted in chemoresistance [33].

HNRNPLL, a tissue-specific heterogeneous nuclear ribonucleoprotein, has been shown to be related to progression of colorectal cancer (CRC). Sakuma et al. [29] found that HNRNPLL suppressed metastasis via EMT. Interestingly, another one of their studies [27] suggested that HNRNPLL stabilized the transcripts of DNA replication regulators (PCNA, RFC3, and FEN1), thus accelerating the proliferation of CRC cells. It has been reported that HNRNPLL could reduce T cell accumulation in lymphoid tissues [34], which was consistent with the findings of our study. This indicates that an SF may impact tumor development and prognosis in multiple mechanisms. We demonstrated that NOVA1, HNRNPC, HNRNPLL, and RBM4 were independent prognostic factors for glioma, and their expression was verified by western blotting analysis. In contrast, NOVA1 is considered to be an oncogene and a prognostic factor in multiple malignant tumors, including CRC [35], thyroid cancer [36], breast cancer [37], melanoma [30], osteosarcoma [38], and gastric cancer [31, 39]. This phenomenon has also been observed for other SFs. We speculate that, unlike the effect of HNRNPLL in CRC, the overexpression of these four SFs may facilitate glioma metastasis but suppress glioma cell proliferation. However, considering the biological characteristics of glioma, it is difficult to study carcinogenesis and construct models to better predict patient prognosis. Notably, SFs suppressing cell proliferation also exist in other types of tumors [40–42]. However, the innate significant heterogeneity of glioma samples may lead to different results. Consequently, more studies are warranted to further verify this result and uncover the mechanism behind this phenomenon.

In conclusion, this study screened and analyzed AS events in glioma using TCGA database. We created prognostic signatures associated with 42 AS events, which displayed favorable performance in predicting prognosis in glioma patients. We also discovered that the SFs NOVA1, HNRNPC, HNRNPLL, and RBM4 are independent risk factors that affect the prognosis and immune cell infiltration of patients with glioma. Certain limitations should also be noted. First, our research was based on TCGA and UCSC Xena databases and was not verified using other independent databases. Second, the four prognosis-related SFs identified in our results may differ in terms of their expression in other tumor types, and the real mechanism behind their association with survival and immune infiltration should be further verified. In summary, our research illustrates the value of AS events and AS-related genes in glioma and presents a survival-related molecular subtype-SF interaction network, which provides a bioinformatics basis for subsequent study of related mechanisms.

## Data Availability

The data used in this study are available from the corresponding authors upon request.
